# Relationship between the Prevalence of Thyroid Nodules and Metabolic Syndrome in the Iodine-Adequate Area of Hangzhou, China: A Cross-Sectional and Cohort Study

**DOI:** 10.1155/2014/675796

**Published:** 2014-08-17

**Authors:** Junhua Yin, Changchun Wang, Qin Shao, Dihong Qu, Zhenya Song, Pengfei Shan, Tao Zhang, Jun Xu, Qin Liang, Songzhao Zhang, Jian Huang

**Affiliations:** ^1^Department of International Health Care Center, The Second Affiliated Hospital ZheJiang University College of Medicine, Hangzhou, Zhejiang 310009, China; ^2^Department of Endocrinology and Metabolism, The Second Affiliated Hospital Zhejiang University College of Medicine, Hangzhou, Zhejiang 310009, China; ^3^Department of Ultrasound, The Second Affiliated Hospital Zhejiang University College of Medicine, Hangzhou, Zhejiang 310009, China; ^4^Department of Clinical Laboratory, The Second Affiliated Hospital Zhejiang University College of Medicine, Hangzhou, Zhejiang 310009, China; ^5^Department of Oncology, Cancer Institute, The Second Affiliated Hospital Zhejiang University School of Medicine, 88 Jiefang Road, Hangzhou, Zhejiang 310009, China

## Abstract

*Objective*. The association between thyroid nodule (TN) prevalence and metabolic syndrome (MetS) has only rarely been examined in iodine-adequate areas and needs further clarification. We investigated correlations between MetS and TN prevalence in the iodine-adequate area of Hangzhou, China. *Material and Method*. A cross-sectional study that screened and recruited individuals for cohort research 3 years later. The 13522 subjects (8926 men, 4596 women) were screened in 2009 for all MetS components, thyroid ultrasound (US), and thyroid function. Cohort research recruited 1610 subjects who were screened in both 2009 and 2012, of whom 1061 underwent follow-up research. *Results*. The prevalence of TN was higher in the MetS (+) group than in the MetS (−) group (*χ* ^2^ = 69.63, *P* < 0.001) and higher in women than in men (*χ* ^2^ = 11.65, *P* = 0.001). Waist circumference (WC) was positively related to the prevalence of TN (OR = 1.022, *P* < 0.001). Individuals with greater WC in 2009 were more likely to suffer from TN in 2012 (RR = 1.434, *P* = 0.024). Elevated triglyceride level was a risk factor for developing new TN (RR = 1.001, *P* = 0.035). *Conclusion*. Both greater WC and elevated triglycerides are risk factors for new TN in this iodine-adequate area in China.

## 1. Introduction

According to the American Thyroid Association (ATA) Guidelines [1], a thyroid nodule (TN) is a discrete lesion within the thyroid gland that is radiologically distinct from the surrounding thyroid parenchyma. Palpable thyroid nodules occur in 4–8% of the population, but nodules incidentally found by ultrasound (US) have a prevalence of 13–67% [2]. About 5% of the clinically apparent thyroid nodules are malignant [2], while nonpalpable nodules have the same risk of malignancy as palpable nodules of the same size [3]. Actually, the proportion of malignant nodules is higher, which reaches 5–15% [4, 5]. With the wider use of neck imaging, the incidence of thyroid nodules has increased by up to 67% in recent decades, which means that we might face increasing prevalence of thyroid cancer in the near future. We are therefore obliged to investigate the relevant risk factors of thyroid nodules.

Previous studies have shown the associations between TN and iodine level [6, 7] and thyroid function parameters [8, 9]. It is reported that the level of serum thyroxine is higher in individuals with TN than those without TN, and the level of serum thyroid stimulating hormone (TSH) is lower in individuals with TN [8, 9]. With the development of westernized lifestyles in China, especially in its eastern regions, obesity and metabolic diseases have already become serious health problems. Increased attention has been given to the relationship between thyroid function and the components of metabolic syndrome (MetS). It has been reported that free tetraiodothyronine (FT4) is significantly associated with the components of MetS [10, 11]. TSH is an independently influential factor for increased triglyceride (TG) level [12]. Insulin resistance (IR) is considered the key pathophysiological mechanism of all these phenomena [13].

Are the components of MetS then independently influential factors for TN? Previous studies have investigated thyroid function and morphological alterations in relation to obesity [14] or glucose metabolism [15]. Our present study is a large-sample cross-sectional and cohort study to show the relationship between the prevalence of TN and thyroid function and MetS components aged 20 to 90. It also describes the prevalence and the age- and sex-specific distribution of TN in Hangzhou, Zhejiang Province, China. In Hangzhou, iodine prophylaxis was started in 1995. The median of Hangzhou residents' urinary iodine excretion is 178.80 *μ*g/L in 2010 [16], which indicates this is an iodine-adequate area according to the WHO criteria [17].

## 2. Subjects and Methods

### 2.1. Study Subjects

The samples of the present single-center study were selected from the International Health Care Center of the Second Affiliated Hospital of Zhejiang University College of Medicine. This was a cross-sectional and cohort study, which was approved by the ethics committee of the hospital. All participants provided informed consent. From January to December 2009, a total of 19622 participants (12814 men, 6808 women) voluntarily underwent general health screening in our hospital, which included thyroid US, the parameters of thyroid function, thyroid autoantibodies, and anthropometric and laboratory data. The participants came from various occupations (factory workers, medical workers, teachers, businessmen, athletes, bank clerks, civil servants, and housewives). The medical history of each participant was obtained from a questionnaire, including previous diagnoses of diabetes, hypertension, thyroid diseases, liver diseases, urticaria, and lipid abnormality, whether the subject was taking antidiabetic and/or antihypertensive medication and/or lipid-lowering drugs, and whether the subject had had thyroid therapy performed including medicine and/or surgery or radiotherapy for the head and neck. The inclusion criteria were aged between 20 and 90 years. Participants with any of the following characteristics were excluded from the study: (a) individuals with history of thyroid diseases; (b) individuals with history of thyroid therapy at any time including medicines (L-thyroxine, antithyroid gland medicine), operation, or radiotherapy for head and neck; (c) individuals with some chronic diseases (hepatic or renal dysfunction, cardiac failure, and urticaria); (d) pregnant or lactating women; (e) individuals with significant mental or neurological disorders (depression, epilepsy, and schizophrenia); (f) individuals with history of other endocrine diseases or autoimmune diseases; (g) individuals with history of cancer(s); (h) individuals with iodinated contrast material exposure in the previous 6 months; (i) individuals with history of taking amiodarone. In the end, 13522 participants (8926 men, 4596 women) were included; of these, 1610 individuals who underwent health screening mentioned above both in 2009 and in 2012 (1109 men, 501 women) were recruited for cohort research. Those participants without thyroid disease at the beginning of the cohort study, 1061 in number (772 men, 289 women), underwent a follow-up study. And 492 persons with TN were followed up for observation of the evolution of TN. The remaining 57 persons with thyroid disease such as overt or subclinical hyperthyroidism and hypothyroidism and high thyroid autoantibody titers were excluded.

### 2.2. Methods

#### 2.2.1. Definitions

Euthyroidism was defined as TSH (reference range, 0.35–4.60 mIU/L), free triiodothyronine (FT3: reference range, 3.50–6.50 pM), and FT4 (reference range, 8.90–20.60 pM) within the normal reference range. In our hospital the reference range of total tetraiodothyronine (TT4) is 60.0 to 165.0 nM and total triiodothyronine (TT3) is 1.23 to 3.02 nM, thyroid peroxidase antibodies (TPO-Ab) is 0 to 34.00 IU/mL, and thyroglobulin antibodies (TG-Ab) is 0 to 115.00 IU/mL.

Diagnoses of MetS in our study were made according to the International Diabetes Federation (IDF) criteria in 2006, which were defined as central obesity (defined as a WC ≥ 90 cm and ≥ 80 cm for Chinese men and women, respectively, with other values for other ethnicities) plus any two of the following four factors: (1) raised TG level (≥1.7 mM, 150 mg/dL) or specific treatment for this lipid abnormality; (2) reduced high-density lipoprotein cholesterol (HDL-c) (<1.03 mM, 40 mg/dL in men and <1.29 mM, 50 mg/dL in women) or specific treatment for these lipid abnormalities; (3) raised blood pressure (BP) (systolic ≥ 130 mmHg or diastolic ≥ 85 mmHg) or treatment of previously diagnosed hypertension; (4) raised fasting plasma glucose (FPG ≥ 5.6 mM, 100 mg/dL) or previously diagnosed type 2 diabetes [18].

#### 2.2.2. Anthropometric Measurements

Each participant underwent a detailed physical examination, including the measurement of BP and WC. WC was measured on bare skin with a folding tape in a horizontal plane, midway between the inferior margin of the ribs and the superior border of the iliac crest in centimeters to the nearest 0.1 cm. Systolic blood pressure (SBP) and diastolic blood pressure (DBP) were measured twice with an automatic blood pressure monitor (Kenz PM SP-1, Suzuken Co. Ltd., Nagoya, Japan) on the right upper arm with the participant seated quietly for at least 5 min; the lower value of measurement was used in the report.

#### 2.2.3. Thyroid Ultrasound Scan

Thyroid was assessed by two experienced physicians using B-mode US imaging (Aloka Alpha10; Aloka Co, Ltd, Tokyo, Japan) with a 10-MHz linear array probe. The physicians performing the imaging were registered US doctors who had a professional certificate for US measurement awarded by the Ministry of Health of China and were blinded to the clinical and laboratory results.

#### 2.2.4. Laboratory Analysis

Venous blood samples were collected using routine methods between 0700 h and 0930 h after overnight fasting. TT4, FT4, TT3, FT3, and TSH were measured with an Advia Centaur XP immunity analyzer (Siemens Corporation) and thyroid autoantibodies (TPO-Ab and TG-Ab) were measured with a Roche electrochemical immunity analyzer (E170). FPG, fasting insulin, TG, and HDL-c were measured with an Olympus AU4500 automatic chemistry analyzer (Olympus Corporation, Tokyo, Japan). The homeostasis model assessment (HOMA) index for IR (HOMA-IR) was calculated as fasting insulin (mU/L) times FPG (mmol/L) divided by 22.5.

#### 2.2.5. Statistical Analysis

All continuous data were expressed as the mean ± SD. The SPSS 17.0 software package (SPSS Inc., Chicago, IL) was used for statistical analysis. Continuous data were used by independent-sample *t*-tests and category data were used by *χ* ^2^ tests for percentages. The age trend of TN prevalence was evaluated by *χ* ^2^ tests to assess tendency.

In the cross-sectional study, multivariate binary logistic regression analysis using the enter method was applied to assess risk factors for the prevalence of TN (which was a dependent variable), with sex, age, SBP, DBP, WC, HOMA-IR, insulin, FPG, HDL-c, TT4, FT4, TT3, FT3, and TSH as independent variables. Univariate binary logistic regression analysis using diverse covariates as adjustment factors was performed to assess the impact on different independent variables. In the cohort study, multivariate binary logistic regression analysis used the forward LR or backward LR method to assess the risk factors for forming TN. All statistical tests were two-tailed, and *P* values <0.05 were considered significant.

## 3. Results

### 3.1. The Prevalence of TN

**Figure 1:**
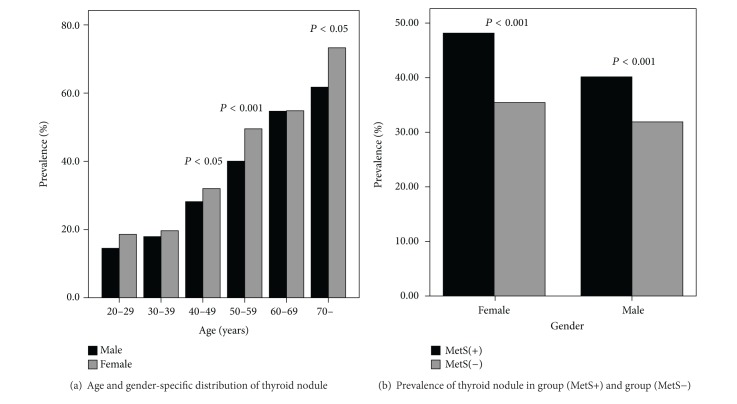
Prevalence of thyroid nodules. (a) The prevalence of thyroid nodules for women was significantly higher than for men in the 40 to 49 age range, 50 to 59 age range, and the age above 70 (*χ* ^2^ = 8.904, *P* = 0.003; *χ* ^2^ = 29.044, *P* < 0.001; *χ* ^2^ = 4.631, *P* = 0.031, respectively). The prevalence of thyroid nodules increased along with increasing age (trend *χ* ^2^ test: *χ* ^2^ = 515.22 for men, *P* < 0.001; *χ* ^2^ = 921.56 for women, *P* < 0.001). (b) The prevalence of thyroid nodule in the MetS (+) group was higher than in the MetS (−) group for both sexes together (41.71% (1157/2774) versus 33.23% (3572/10748), *χ* ^2^ = 69.63, *P* < 0.001) and for men and women separately (female: 48.15% (260/540) versus 35.43% (1437/4056), *χ* ^2^ = 33.10, *P* < 0.001; male: 40.15% (897/2234) versus 31.90% (2135/6692), *χ* ^2^ = 50.80, *P* < 0.001).

The study population characteristics are depicted in Figure 1(a). As noted, the cross-sectional study included a total of 13522 participants (8926 men and 4596 women). The prevalence of TN was 34.97% (33.97% for men and 36.92% for women). The standardized morbidity rate of TN was 33.70% (31.82% for men and 35.35% for women), which was calculated from the population distribution in China in 2009. The prevalence of TN for women was statistically higher than for men in the age ranges 40 to 59 and >70 and was significantly higher in the age range 50 to 59 (49.54% versus 40.04%, *χ* ^2^ = 29.044, *P* < 0.001), and so was the prevalence over all ages (*χ* ^2^ = 11.651, *P* = 0.001). Furthermore, the prevalence of TN increased along with increased age (trend *χ* ^2^ test: *χ* ^2^ = 1200.33 for both sexes, *P* < 0.001; *χ* ^2^ = 515.22 for men, *P* < 0.001; *χ* ^2^ = 921.56 for women, *P* < 0.001).

### 3.2. The Relationship between TN and MetS

As shown in Figure 1(b), the number of MetS (+) participants, who were selected according to the standard of the IDF, was 2774 (2234 men, 540 women), and the prevalence of TN was 41.71% (1157/2774). The number of MetS (−) participants was 10748 (6692 men, 4056 women), and the prevalence of TN was 33.23% (3572/10748). The prevalence of TN in the MetS (+) group was higher than that in the MetS (−) group (*χ* ^2^ = 69.63, *P* < 0.001).

### 3.3. The Relationship between TN and Metabolic Components

As shown in Table 1, there was a significant correlation between the prevalence of TN and SBP, DBP, WC, insulin, and HOMA-IR using univariate binary logistic regression analysis. The prevalence of TN was not related to HDL-c in the crude model, but it was significantly related to it after adjustment by sex, age, TT4, and TSH. The prevalence of TN was significantly related to FPG in the crude model but no longer significant after the above adjustment. With multivariate binary logistic regression analysis, thyroid nodule was defined as a dependent variable, while sex, age, SBP, DBP, WC, HOMA-IR, FPG, HDL-c, TT4, FT4, TT3, FT3, and TSH were independent. The results were almost consistent with the univariate binary logistic regression analysis when either the enter method or the forward LR method was used (data not shown); the prevalence of TN was significantly related to sex, age, and WC (WC: OR = 1.022, 95% CI 1.015–1.029, *P* < 0.001). WC was an independent risk factor for the prevalence of TN.

### 3.4. The Relationship between TN and Metabolic Components in Autoimmune Thyroiditis

In the current cross-sectional study, the individuals with autoimmune thyroiditis were considered as an independent group. According to the inclusion and exclusion criteria of this study and the diagnostic criteria of autoimmune thyroiditis [19], 989 individuals were included in this group; of these, 332 individuals were autoimmune thyroiditis with nodule and 657 ones were autoimmune thyroiditis without nodule.

In the group of autoimmune thyroiditis, the prevalence of TN was not related to any metabolic component whether univariate binary logistic regression analysis or multivariate binary logistic regression analysis was used (data not shown).

The morbidity of Mets was 26.09% in autoimmune thyroiditis positive group and 17.37% in autoimmune thyroiditis negative group (*χ* ^2^ = 46.25, OR = 1.678, 95% CI, 1.444~1.951, and *P* < 0.001). Then, a multivariate binary logistic regression analysis was used: the impacts of autoimmune thyroiditis on Mets were FBG (OR = 0.538, 95% CI, 0.538~0.828, and *P* < 0.001), TG (OR = 0.780, 95% CI, 0.651~0.935, and *P* = 0.007), and HDL (OR = 5.465, 95% CI, 4.621~6.464, and *P* < 0.001). There were no significant impacts on BP and WC. The morbidity of TN was 33.57% in autoimmune thyroiditis positive group and 34.97% in autoimmune thyroiditis negative group (*χ* ^2^ = 0.799, *P* = 0.371).

### 3.5. Results of 3-Year Follow-Up

The 1061 participants without thyroid disease in 2009 (772 men, 289 women) were enrolled in the cohort study. The results of follow-up were shown in Table 2. For both men and women the prevalence of TN in 2012 was higher than that in 2009 (43.13% versus 33.97% for men, *P* < 0.001; 42.56% versus 36.92% for women, *P* = 0.006). We compared the mean value by age and by WC between the follow-up group and the total population group, which included 13522 individuals in 2009. There were no statistical differences (age: 48.02 ± 9.04 versus 48.32 ± 9.84, *t* = 1.008, and *P* = 0.293; WC: 85.94 ± 9.24 versus 85.67 ± 9.40, *t* = 0.884, and *P* = 0.377). The proportion of women was statistically different between the follow-up group and the total population group (the proportion for women: 27.24% versus 33.99%, c). Like the prevalence of TN in 2009, the morbidity rates of TN in 2012 for both men and women gradually increased along with increasing age.

We then studied whether the MetS components of the participants in 2009 were responsible for the prevalence of TN in the follow-up. We found that the individuals with larger WC in 2009 were more inclined to suffer from TN in 2012 (RR = 1.422, *P* = 0.007) (Table 3). We then analyzed whether the dynamic changes in the MetS components contributed to the forming of TN. The gradually increasing TG level was a risk factor for new TN (RR = 1.001, *P* = 0.035). It was also consistent whether the forward LR method or the backward LR method was used in the multivariate binary logistic regression analysis (Table 4). The number of individuals with diabetes was 38 in 2009. 18 of them developed thyroid nodule in 2012. And 466 of the left individuals without diabetes (with a number of 1023) developed thyroid nodule. There was no significant difference between the group with diabetes and the group without it (*χ* ^2^ = 0.049, *P* = 0.852).

Among all individuals without thyroid disease in 2009, it was eventually found in the process of follow-up that 7 out of 1061 participants suffered from thyroid cancer (0.66%). We further reviewed the 492 participants with TN in 2009. Two cases (0.41%) were diagnosed pathologically as thyroid cancer before 2012.

## 4. Discussion

Our study showed that the prevalence of TN in MetS (+) individuals was higher than that in MetS (−) individuals and that WC was an independent risk factor for TN. Recently, more attention has been paid to the correlation between MetS or its components and thyroid function and its volume [20]. Rezzonico et al. [20] prove that the higher circulating levels of insulin cause increased thyroid proliferation. The clinical manifestations are enlarged thyroid volume and the formation of nodules. They thus conclude that the thyroid gland might be one of the “victims” of the insulin resistance syndrome. As defined in the IDF criteria, central obesity is the core of MetS, and IR is the key mechanism of MetS [21]. Van Keymeulen et al. [22] show that insulin receptors, once expressed at a sufficient level, signal a (co)mitogenic cascade as efficiently as insulin-like growth factor-1 (IGF-1) receptors. Obesity is associated with increased free or bioavailable IGF-1. According to Table 1, we showed by univariate logistic analysis that both plasma insulin level and HOMA-IR were correlated with TN. Therefore, the correlation between WC and TN might be mediated by IR.

Huggett et al. [23] consider that individuals with MetS have excessive activity of the sympathetic nerves. Most individuals with obesity exhibit increased activity of the sympathetic nerves too [24]. Grassi et al. [25] confirm that the activity of sympathetic nerves is stimulated by insulin. The sympathetic nerve activity in individuals with central obesity is higher than that in individuals with peripheral obesity [26]. Elevation in the serum level of FT3, which is one of the calorific hormones, is probably a compensatory mechanism for central accumulation of fat [27]. The expression of lipogenic enzyme is mediated by T3 through the *α* subunit of the thyroxine receptor, which strengthens the accumulation of lipid droplets and is significantly responsible for forming and maintaining mature fat [28]. Therefore, sympathetic nerves passageway for central obesity is related to thyroid.

As for the group of autoimmune thyroiditis, the prevalence of TN was not related to any metabolic component. This result indicates that autoimmune pathway might be one of mechanisms of TN forming, and autoimmunity might play a more important role than metabolism does in the TN forming process in this group. The size of the group of autoimmune thyroiditis might be one of the probable factors that influence the conclusion.

In the current cohort study, we found both larger WC and gradually increasing TG level were risk factors for new TN. It is not difficult to understand the relation of the two risk factors. Individuals with increasing TG levels in plasma are more inclined to develop central obesity and then suffer from larger WC. Both our cross-sectional study and cohort study demonstrated that the larger WC was an independent risk factor for TN. As for the morbidity rate of thyroid cancer in the follow-up process, the size of the samples was too small to confirm that. A large-sample study is therefore recommended to confirm the correlation between thyroid cancer and MetS.

The previous study suggested that the TN prevalence is higher in women than in men [1]. In the current study the difference existed mainly in the age range 40 to 59. As we all know, women in this age range were menopausal. This phenomenon suggested that disorder of internal sex hormones might be responsible for the formation of TN. The wave of sex hormone binding globulin (SHBG) might play an important role in this process [29, 30]. Our data indicates that the prevalence of TN in both men and women increases gradually with age, which is consistent with previous studies [31, 32]. Therefore, age is another risk factor for TN. Middle and elderly women have higher incidence of TN than men.

There were some limitations to this study. Firstly, there was lack of data regarding the mean volume of the thyroid glands and the size of thyroid nodules. Secondly, no data from cytological and/or histopathological analyses of each TN was available for many participants as they refused fine needle aspiration investigations. Thirdly, the participants did not come from the randomly selected samples, who voluntarily underwent general health screening and voluntarily participated in this cross-sectional study. However, with the large sample size and broad distribution of occupations in this study, our data could, to some degree, represent the general population. Although the present study did not concern the other ethnicities, we researched some similar results which had been reported in Korea [11], Argentina [20], and Turkey [33]. Therefore, this finding might be applicable for other ethnicities.

## 5. Conclusion

The prevalence of TN in the MetS (+) group is higher than in the MetS (−) group. There is a significant relationship between WC and the prevalence of TN, independently of age, gender, blood pressure, fasting glucose, HDL, insulin, and HOMA-IR. Both the larger WC and the increasing TG are risk factors for forming thyroid nodules. The results of this study indicate that maintaining WC and TG in a suitable range might be helpful in decreasing the prevalence of TN.

## Figures and Tables

**Table 1:** Univariate binary logistic regression analysis: dependent variable is thyroid nodule.

	Model	*β*	*P* value	OR	95% C.I. for OR	
SBP	1	0.013	0.000	1.014	1.011	1.016
	2	0.005	0.001	1.005	1.002	1.007
	3	0.004	0.010	1.004	1.001	1.007

DBP	1	0.014	0.000	1.014	1.011	1.017
	2	0.007	0.000	1.007	1.004	1.010
	3	0.006	0.001	1.006	1.002	1.010

WC	1	0.020	0.000	1.020	1.016	1.024
	2	0.018	0.000	1.018	1.014	1.023
	3	0.020	0.000	1.020	1.014	1.025

FBG	1	0.006	0.000	1.006	1.004	1.007
	2	0.001	0.324	1.001	0.999	1.003
	3	0.001	0.609	1.001	0.998	1.003

Insulin	1	0.007	0.032	1.007	1.001	1.014
	2	0.012	0.001	1.012	1.005	1.019
	3	0.011	0.007	1.011	1.003	1.018

HOMAIR	1	0.002	0.000	1.002	1.001	1.004
	2	0.002	0.001	1.002	1.001	1.004
	3	0.002	0.010	1.002	1.000	1.004

TG	1	0.000	0.792	1.000	1.000	1.000
	2	0.000	0.791	1.000	1.000	1.000
	3	0.000	0.836	1.000	1.000	1.000

HDL	1	−0.002	0.130	0.998	0.995	1.001
	2	−0.004	0.012	0.996	0.993	0.999
	3	−0.004	0.030	0.996	0.992	1.000

Model 1, crude; Model 2, after adjustment for age and sex; Model 3, after further adjustment for TT4 and TSH.

SBP: systolic blood pressure; DBP: diastolic blood pressure; WC: waist circumference; FBG: fasting blood glucose; HOMA-IR: homeostasis model assessment (HOMA) index for insulin resistance; TG: triglyceride; HDL: high-density lipoprotein.

**Table 2:** The results of follow-up for participants without thyroid diseases in 2009.

Results in 2012	Male		Female	
	*n*	%	*n*	%
Normal	417	54.02	149	51.56
TN	333	43.13	123	42.56
Cancer	5	0.65	2	0.69
DG	6	0.78	0	0.00
HT	10	1.30	15	5.19
SAT	1	0.13	0	0.00

Total	772		289	

All of the results were based on B-mode ultrasound scanning.

TN: thyroid nodule; DG: diffuse goiter; SAT: subacute thyroiditis; HT: Hashimoto's thyroiditis.

**Table 3:** Univariate binary logistic regression analysis: dependent variable is thyroid nodule in 2012, and independent variables are the status of the metabolic components, Mets, HOMA-IR, and Insulin in 2009. (Method: Enter).

Independent variable	*β*	*P* value	RR	95% C.I. for RR	
Hypertension	0.288	0.031	1.333	1.027	1.731
Hyperglycemia	−0.008	0.960	0.992	0.714	1.377
Higher TG	−0.263	0.073	0.769	0.577	1.024
Lower HDL	0.053	0.726	1.055	0.782	1.423
Larger WC	0.352	0.007	1.422	1.100	1.839
MetS	0.136	0.405	1.145	0.832	1.576
HOMA-IR	−0.002	0.337	0.998	0.993	1.002
Insulin	−0.010	0.374	0.990	0.969	1.012

All of the metabolic components in 2009 were transferred into qualitative data according to the IDF criteria; MetS was defined according to the IDF criteria as well. All independent variables were adjusted by age and sex. Hypertension lost statistical significance (*P* = 0.093) in multivariate binary logistic regression analysis when hypertension and WC were taken as independent variables.

TG: triglyceride; HDL: high density lipoprotein; WC: waist circumference; HOMA-IR: homeostasis model assessment (HOMA) index for insulin resistance; MetS: metabolic syndrome.

**Table 4:** Multivariate binary logistic regression analysis: dependent variable is thyroid nodule in 2012, and independent variables are age, sex, and the *D* values of SBP, DBP, WC, FBG, TG, and HDL (method: forward stepwise LR or backward stepwise LR).

	*β*	*P* value	RR	95% C.I. for RR	
Gender	0.055	0.000	1.057	1.040	1.073
*D* value of TG	0.001	0.035	1.001	1.000	1.002
Constant	−2.759	0.000	0.063		

*D* value of TG was calculated as the TG value in 2012 minus the TG value in 2009, and the differences of SBP, DBP, WC, FBG, and HDL were calculated in the same way. The results of forward stepwise LR and backward stepwise LR were absolutely consistent.
